# Neonicotinoid-contaminated pollinator strips adjacent to cropland reduce honey bee nutritional status

**DOI:** 10.1038/srep29608

**Published:** 2016-07-14

**Authors:** Christina L. Mogren, Jonathan G. Lundgren

**Affiliations:** 1 USDA-ARS, 2923 Medary Ave, Brookings, SD 57006, USA

## Abstract

Worldwide pollinator declines are attributed to a number of factors, including pesticide exposures. Neonicotinoid insecticides specifically have been detected in surface waters, non-target vegetation, and bee products, but the risks posed by environmental exposures are still not well understood. Pollinator strips were tested for clothianidin contamination in plant tissues, and the risks to honey bees assessed. An enzyme-linked immunosorbent assay (ELISA) quantified clothianidin in leaf, nectar, honey, and bee bread at organic and seed-treated farms. Total glycogen, lipids, and protein from honey bee workers were quantified. The proportion of plants testing positive for clothianidin were the same between treatments. Leaf tissue and honey had similar concentrations of clothianidin between organic and seed-treated farms. Honey (mean±SE: 6.61 ± 0.88 ppb clothianidin per hive) had seven times greater concentrations than nectar collected by bees (0.94 ± 0.09 ppb). Bee bread collected from organic sites (25.8 ± 3.0 ppb) had significantly less clothianidin than those at seed treated locations (41.6 ± 2.9 ppb). Increasing concentrations of clothianidin in bee bread were correlated with decreased glycogen, lipid, and protein in workers. This study shows that small, isolated areas set aside for conservation do not provide spatial or temporal relief from neonicotinoid exposures in agricultural regions where their use is largely prophylactic.

Worldwide pollinator declines have sparked controversy and debates regarding the specific causes of these declines, especially in managed honey bees (Apidae: *Apis mellifera*)^1^,^2^,^3^. The phenomenon termed colony collapse disorder (CCD) is hypothesized to result from a number of factors including diseases and parasites, in-hive and environmental pesticide exposure, reduced access to quality forage, and changing cultural practices of beekeeping^4^,^5^,^6^. Insecticide exposure, especially to the neonicotinoids, has garnered much attention in recent years, prompting a two-year moratorium by the European Union on their use in flowering crops^1^, reductions on their use in corn and soy in Ontario, Canada^2^, and the United States Environmental Protection Agency to enact guidelines limiting pollinator exposures in treated cropland.

However, widespread and prophylactic use of neonicotinoids and other pesticides in the United States^7^,^8^ as a result of policy-driven changes in agronomic practices^9^, particularly in the Upper Midwest, has resulted in decreased access to forage^10^ (with the removal of vegetation surrounding fields to maximize crop production) and increased risk of neonicotinoid exposure for the majority of the nation’s honey bees during the months of honey production.

Understanding the environmental fate of neonicotinoids improves our perceptions of environmental risks posed by this class of insecticides. Only 2–20% of the active ingredient on treated crop seeds is taken into the developing plant^11^, and recent data suggests that the remaining majority of the compound is not staying within cropland^12^,^13^,^14^,^15^,^16^,^17^,^18^. Soil half-lives vary greatly between chemical forms and they may remain for years after initial application (e.g. thiamethoxam: 25–100 days^19^, clothianidin: 148–1,155 days^20^). Clothianidin, thiamethoxam, and imidacloprid have been detected in major waterways and wetlands that drain agricultural areas^14^,^16^, implicating runoff events in creating long-term exposure scenarios for aquatic organisms. Residues have also been documented in non-target plants adjacent to treated areas^12^,^13^,^15^,^18^, which serve as critical forage for honey bees and other pollinators. Pesticides are often detected in nectar, honey, pollen, and beeswax^17^, indicating that contaminated forage provides a potential route of exposure to bees.

With some exceptions, researchers have had difficulty showing field-relevant exposures to neonicotinoids having an appreciable effect on honey bee colony performance^4^,^21^, and the levels that have been detected in plants and bee products are arguably not high enough to induce acute mortality in honey bees (thiamethoxam: oral LD_50_ = 5 ng/bee, clothianidin: oral LD_50_ = 4 ng/bee^22^, but see Henry, *et al*.^21^]). However, sublethal effects have been observed in bees at field realistic exposures resulting in impaired foraging behavior^23^,^24^, decreased reproductive capacity^23^,^25^,^26^,^27^, and synergistic interactions with other stressors such as pathogens^28^,^29^. A typical approach to addressing the effects of pesticides on honey bees has mostly focused on endpoints of colony performance, as honey bees are managed as colonies for honey production and pollination services. High annual losses in the United States^30^ have been difficult to attribute to neonicotinoids specifically, but sublethal impairment to exposed foragers as measured by nutrient status may be useful in identifying mechanisms by which losses are indirectly sustained, particularly in winter^31^. Here, we applied measures of glycogen, lipid, and protein levels as proxies for individual bee health, which have been used previously to effectively quantify nutrient status in other insect species (see Pumariño, *et al*.^32^ and references therein).

Initially, the aim of this study was to determine whether increasing forage by planting pollinator strips in a corn and soy dominated region would serve to buffer against harmful effects of plant-incorporated pesticides, specifically the neonicotinoids, with organic corn fields serving as controls. However, when it became apparent that an unintended consequence of planting pollinator strips adjacent to treated cropland meant that they became a source for neonicotinoid exposure, the goal shifted to determine whether pollinator strips were themselves harmful to the bees. The objectives of the present study were to 1) evaluate the presence of clothianidin in pollinator strips near treated cropland, 2) quantify the amounts of clothianidin in floral tissues, and 3) determine whether accumulated levels pose a significant risk to honey bees.

## Results

### Clothianidin concentrations in plant tissues

**Figure 1:**
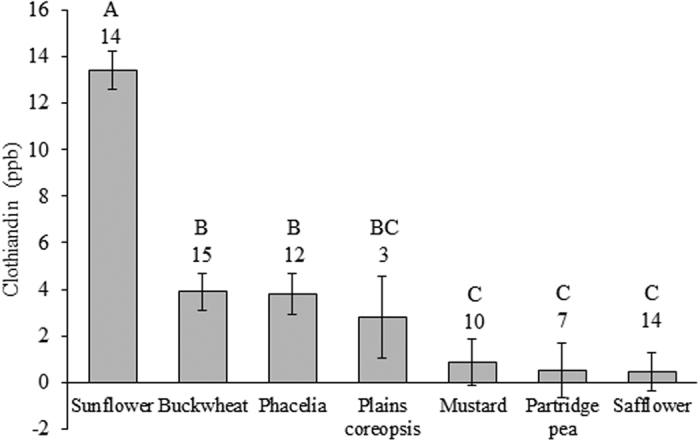
Concentrations of clothianidin in leaf tissues (mean ± SE). Letters above bars show significant differences between plant species and numbers represent the number of site-years in which a particular species was analyzed.

Plant species accumulated different levels of clothianidin in their leaf tissues (F = 26.8, d.f. = 6,61, *P* < 0.001) (Fig. 1). Sunflowers (*Helianthus annuus*), which started blooming in July, accumulated the highest concentrations in their leaves, ranging from 0–81 ppb clothianidin. Buckwheat (*Fagopyrum esculentum*) and phacelia (*Phacelia tanacetifolia*), which start blooming within a month of germination in the spring, had significantly lower concentrations recovered in leaf tissue, ranging from 0–52 ppb and 0–33 ppb, respectively (Fig. 1).

**Figure 2:**
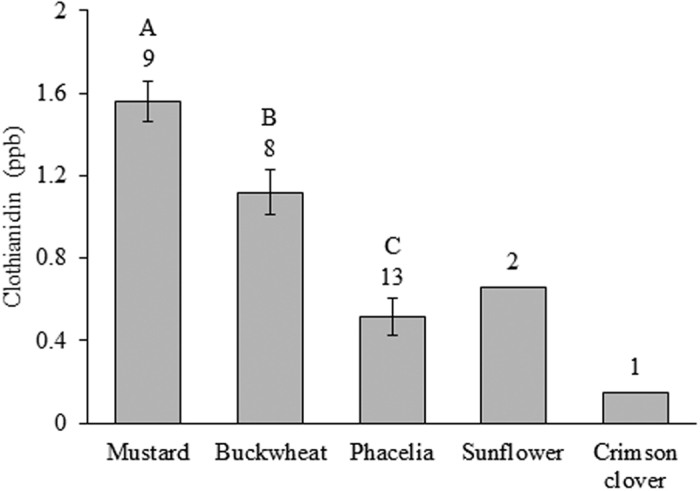
Concentrations of clothianidin in nectar collected by honey bees (mean ± SE). Letters above bars show significant differences between plant species and numbers represent samples analyzed. Sunflower and crimson clover were excluded from analysis due to low replication.

Nectar was only collected in sufficient quantities for analysis from mustard (*Sinapis alba*), buckwheat, phacelia, sunflower, and crimson clover (*Trifolium incarnatum*). However, due to low replication, sunflower and crimson clover were excluded from analysis. Clothianidin was detected in nectar collected by honey bees (Fig. 2), with significant differences between plant species (F = 27.4, d.f. = 2,26, *P* < 0.001).

### Seed-treated versus organic cornfields

Pollinator strips planted adjacent to seed-treated cornfields and untreated organic cornfields had similar clothianidin concentrations in leaf tissues (F = 0.01, d.f. = 1,61, *P* = 0.92), and nectar (F = 0.01, d.f. = 1,26, *P* = 0.91). The proportion of contaminated plants on seed treated and organic farms were not statistically different for any of the plant species tested (Table 1). Clothianidin levels in honey were also similar between seed-treated and organic farms (F = 0.26, d.f. = 1,12, *P* = 0.62), with no differences in residue concentrations between sampling dates (onset of bloom versus post-flowering at the end of the summer; F = 0.40, d.f. = 1,12, *P* = 0.54). Honey levels averaged (±SEM) 6.61 ± 0.88 ppb per hive, approximately seven times greater than concentrations recovered in nectar (0.94 ± 0.09 ppb, averaged across all species). In contrast, bee bread (pollen returned to the hive and stored for food) recovered from the hives located on organic farms had significantly lower levels of clothianidin than bee bread from hives near seed-treated fields (F = 5.40, d.f. = 1,12, *P* = 0.04), with bee bread from seed-treated farms containing nearly twice as much clothianidin (organic: 25.8 ± 3.0 ppb; seed-treated: 41.6 ± 2.9 ppb). Pesticide residues in bee bread were also unaffected by sampling date (F = 0.48, d.f. = 1,12, *P* = 0.50) and there was no significant interaction between sampling date and treatment (F = 1.18, d.f. = 1,12, *P* = 0.30).

### Honey bee health

**Figure 3:**
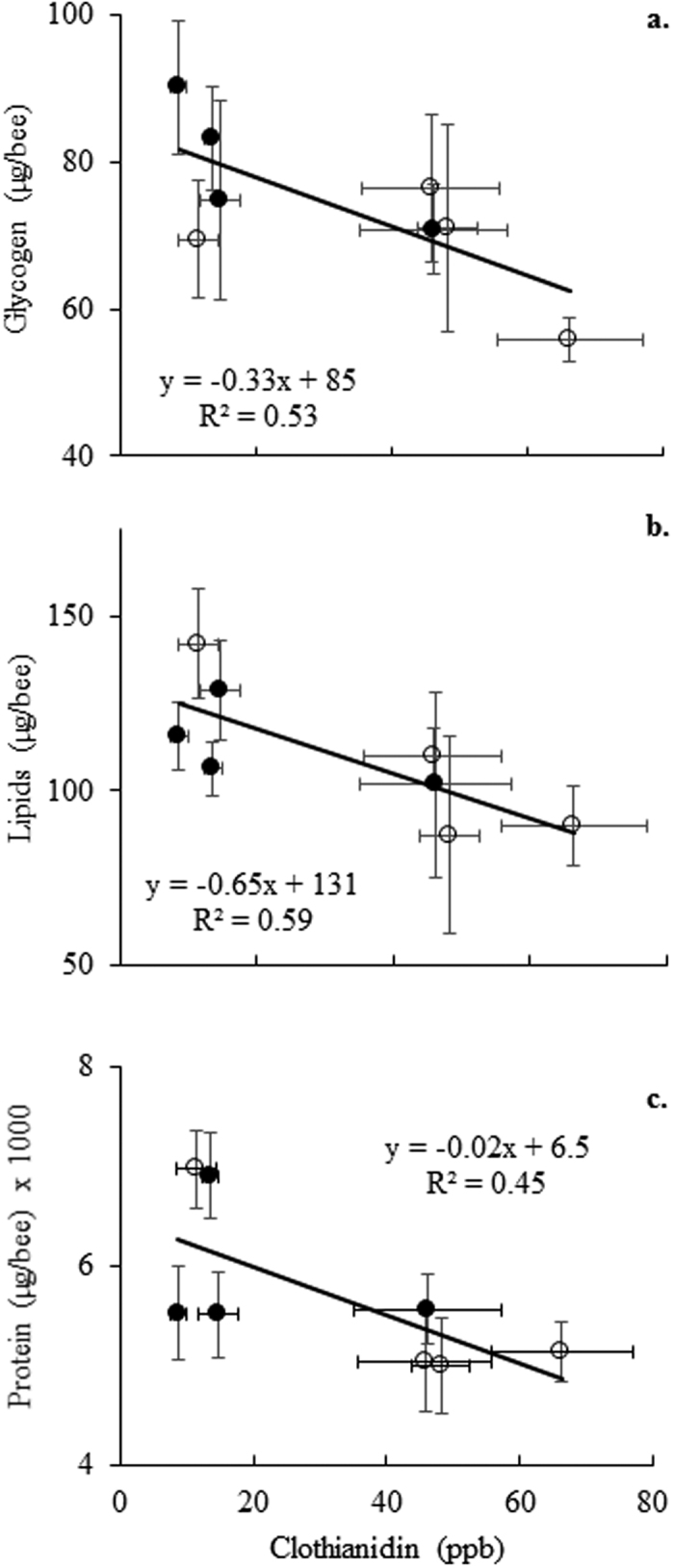
The relationship between clothianidin concentrations in bee bread and total (**a**) glycogen, (**b**) lipids, and (**c**) protein in individual bees, with horizontal and vertical error bars. Black circles are organic farms and white circles are seed-treated farms.

Increasing concentrations of clothianidin in the bee bread collected by honey bees were associated with significant declines in glycogen (F = 6.85, d.f. = 1,6, *P* = 0.04) and lipids (F = 8.73, d.f. = 1,6, *P* = 0.03) in the worker bees at those respective sites, with a negative association in total proteins (F = 4.84, d.f. = 1,6, *P* = 0.07) (Fig. 3). Bees from the hives at organic and treated farms had a similar nutritional status (glycogen: F = 0.82, d.f. = 1,5, *P* = 0.41; lipids: F = 1.32, d.f. = 1,5, *P* = 0.30; protein: F = 0.21, d.f. = 1,5, *P* = 0.67). Clothianidin concentrations of in-hive honey had no effect on honey bee nutritional status (glycogen: F = 0.43, d.f. = 1,6, *P* = 0.54; lipids: F = 2.68, d.f. = 1,6, *P* = 0.15), although a negative trend was observed between honey contamination levels and bee protein quantities (F = 5.08, d.f. = 1,6, *P* = 0.07).

## Discussion

This study indicates that neonicotinoid contamination unintentionally negates, in part, the organic status of small, isolated farms, and questions the benefits of deploying habitat for conservation purposes in the absence of integrated pest management (IPM) practices on cropland. A large proportion of cropland in the region receives neonicotinoid seed treatments^7^,^33^, despite empirical data questioning the benefits of these treatments for producers of a number of commodities^34^,^35^,^36^. An estimated 42 million ha of maize, soybean, and cotton cropland are seed-treated with neonicotinoids in the United States^7^. Regulations dictate that buffer strips between organic and conventional farms be sufficient to prevent unintended application of prohibited substances^37^ and are typically 7.5–9 m wide, though most contaminated planter dust falls within 5 m of the treated field^38^,^39^,^40^. But given the hydrophilic nature of the neonicotinoids (e.g. log K_OW_ clothianidin = 0.7), we hypothesize that spatially isolated organic farms are contaminated by neonicotinoids from sources other than planter dust, and that water^14^,^16^ and soil pathways merit additional study to explain contamination at sites spatially and temporally isolated from treated areas. In short, an unintended consequence of prophylactic use of neonicotinoid seed treatments may be to reduce the benefits of pollinator conservation plans.

In addition to spatially limited forage refugia, we found no temporal relief from non-target contaminated forbs as evidenced by high residue levels recovered in late summer blooming sunflowers (Fig. 1), further suggesting that contamination was not solely from a temporally distinct early season source like planter dust. Thus, it does not appear that plants blooming closer to corn planting necessarily accumulate higher concentrations of clothianidin. Peredovik sunflowers are an ornamental variety, but wild sunflowers are native throughout the upper Great Plains region. This species accumulating the highest concentrations in leaf tissues is contrary to what would be expected given the size of this species relative to the others tested^41^, assuming soil residues were uniform across a particular site. Concentrations were 135 times higher than those reported for a crop sunflower variety treated with thiamethoxam in the R5 stage^36^. However, Bonmatin *et al*.^12^ reported a sharp increase in imidacloprid levels in leaves during flowering for five different cultivated varieties. These differences within a single species may be due to physiological differences between cultivars or the result of abiotic and soil conditions affecting uptake^8^. Regardless, the risk of exposure associated with honey bees foraging on wildflowers is consistently present in space, time, and across flowering forage species within this highly developed agricultural region.

Honey bees are known to have high forage fidelity, and when foraging will only collect resources from a particular plant species^42^, in contrast to other native bee species. It is therefore highly probable that nectar recovered in the crops of honey bees came from the plant species they were collected from. This proved to be an effective and time-saving means for collecting nectar for clothianidin analysis. Residues in buckwheat and phacelia nectar were 72 and 86% less than those recovered in their leaf tissues, respectively, with mustard nectar containing 1.8 times greater concentrations than the leaf tissue. As was the case with leaf tissues, differences were significant among species, indicating physiological mediation by plants with regard to how much clothianidin is taken up from soils. Across plant species, the average concentration detected in nectar was lower here than concentrations recovered from the nectar of neonicotinoid-treated flowering crops (e.g. clover: 2882–2992 ng/g (from a spray application as opposed to a seed treatment)^43^; curcubits: 17.6 ng/g^44^, 10–11 ppb^45^; oilseed rape: 4.2 ng/g^46^, ≤2.24 ppb^47^; ornamental flowers: 21–45 ppm^48^), but similar to those levels reported for wildflowers growing near treated fields (0.10 ng/g^13^, 0–6.5 ng/ml^15^,^27^). The amount of clothianidin in honey from our study was approximately seven times more concentrated than nectar, which would be expected given the average water content of nectar versus honey. However, this also indicates that clothianidin is not breaking down during the process of nectar conversion to honey, a point made by Dively *et al*.^49^ for imidacloprid.

The concentrations we recovered in bee bread were greater at both organic and seed treated farms than those reported elsewhere for contaminated pollen^12^,^13^,^17^,^27^,^45^,^47^. This may be the result of compounding levels of clothianidin from continuous plantings of treated crops year after year in a region dominated by treated corn and soybeans, and minimal levels of breakdown in soils. Further analyses would be needed to quantify clothianidin residues in eastern South Dakota to test this hypothesis.

Using the concentrations we recovered in honey and bee bread, a honey bee worker would need to consume approximately 610 mg of honey or 95 mg of bee bread at one time in order to experience lethality at the LD_50_ level. This exceeds the amount of honey and pollen consumed daily by adult workers (honey: 13–18 mg/day, pollen: 6.5 mg/day) and larvae (honey: 15–29 mg/day, pollen: 1.1 mg/day)^22^, if we assume the same LD_50_ for both. These LD_50_s are largely lacking for bee larvae, and are also strongly affected by the physiological status of the bees tested, so may not be an accurate perception of field-collected bees in our study. Assuming that these LD_50_s are an accurate estimate, acute mortality at the levels observed in our study is unlikely. Nevertheless, clothianidin concentrations in bee bread were associated with declines in nutritional status of the bees under field conditions. While neonicotinoids have been shown to reduce the viability of honey bee queens^25^,^26^, it has been more difficult to detect significant effects on workers and overall colony performance, possibly owing to “superorganism resilience”^50^ or differential detoxification^51^, versus declines found for other eusocial bees and solitary native bees^27^. However, using lethality as an endpoint fails to take into account stress that may be experienced by an individual as a result of exposure, which could weaken an individual enough that additional sublethal stressors, such as disease or other pesticides, lead to mortality or deleterious behavioral modification.

Glycogen and lipids are synthesized and stored in the fat body of insects, and are important as immediate energy sources and to be used during flight and diapause, respectively^52^. Lipids are particularly important in the success of overwintering species^31^. Since CCD was first recognized in 2006, beekeepers have reported 30% annual losses of colonies^30^, many of which occur during the winter months. Significant decreases in individual honey bee lipid storage late in the summer as a result of clothianidin exposure could therefore have significant implications for overwintering success of winter bees and entire colonies. Fat content has also been strongly correlated with reproductive capacity in females of other species^32^,^53^. Should declines in worker lipid content reflect similar declines in queens, this could have negative consequences for egg-laying. Our study documented declines in lipids and glycogen, which also have importance in immunity^52^, and could explain why sublethal neonicotinoid exposures coincide with increases in disease infection rates^28^,^29^.

## Conclusions

The paradox of the present study is that pollinator strips intended to enhance honey bee health in a heavily developed agricultural landscape resulted in declining bee health due to unintended accumulation of clothianidin from adjacent treated corn fields at both organic and conventional farms. Although pollinator strips on organic farms were generally more than 140 m from the nearest treated crop (Supplementary Table S1), this was not far enough to fully isolate these strips from negative seed treatment effects. It has been suggested that any harmful exposures resulting from non-target plant uptake of neonicotinoids would be diluted by the fact that honey bees visit numerous flowers on a single foraging expedition^54^. Dilution would be difficult in highly developed agricultural areas like eastern South Dakota where neonicotinoid seed treatments are ubiquitous. We found that clothianidin uptake was the same at treated and untreated locations, and was present in plant tissues throughout the growing season. The concentrations of clothianidin recovered in bee bread were high enough to impair glycogen and lipid accumulation, with significant implications for overwintering success and reproductive potential of queens. While pollinator strips and uncropped areas have the potential to serve as buffers to pesticide exposures for bees^55^, our results indicate that their placement within the landscape needs to be carefully considered. In all likelihood, reducing bee exposures to these pesticides will require reductions in their use across the landscape and a movement away from prophylactic applications towards more integrated pest management strategies, as has been suggested elsewhere^2^.

## Materials and Methods

### Site selection and preparation

**Figure 4:**
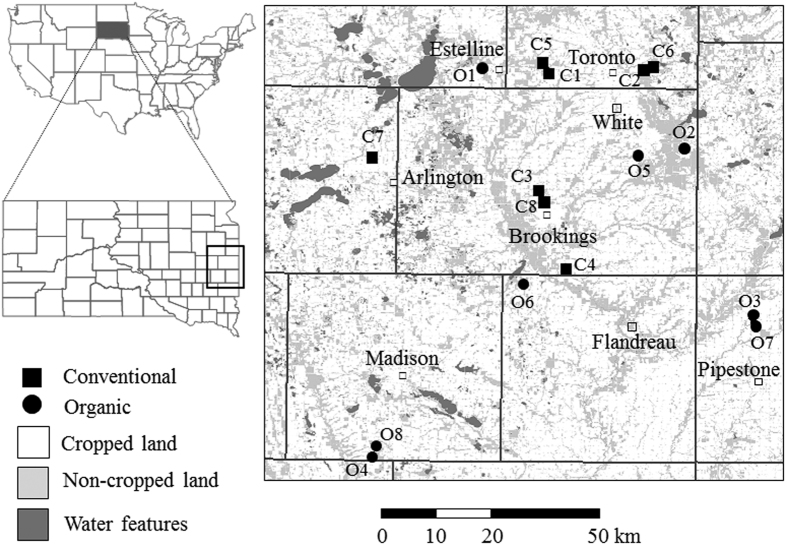
Map of the study region. Field locations were imported into ArcGIS 10.1 (ESRI, Redlands, CA, USA) and mapped using ArcMap (http://desktop.arcgis.com/en/arcmap/). Further site descriptions available in Supplementary Table S1.

Pollinator strips were planted adjacent to 16 corn fields during the 2014 and 2015 growing seasons (n = 8 sites in each year) (Fig. 4, Supplementary Table S1). Half of these were located on certified organic farms (fields in organic cropping practices for at least 5 years) planted with organic field corn (non-Bt and no insecticidal seed treatments), and the other half on conventional farms planted with Bt-transgenic field corn seed-treated with thiamethoxam (as Cruiser) or clothianidin (as Poncho or Acceleron), with 0.25 mg active ingredient/seed (Supplementary Table S1). Within a particular year, sites were located greater than 5 km apart, such that honey bees could not forage between pollinator strips. All corn fields were maintained according to standard agronomic practices (herbicide, fungicide, tillage, and fertilizer regimens) as decided by the producers. Organic fields in the region are typically characterized by higher rates of tillage than conventional fields for weed control and an absence of genetically modified crops or synthetic pesticides or fertilizers. The study region is dominated by corn and soybean row crops and degraded pasture lands, together accounting for 75% of regional land use^10^.

Native and naturalized annual and perennial forb species were included in the pollinator strip mix (Millborn Seeds, Brookings, SD), which was then optimized in 2015 based on 2014 observational data of pollinator visitation and floral preference (Table 2). In addition to including a diversity of colors and floral structure types, this mix contained early and late season flowering forbs to provide honey bee forage for the entire growing season. Pollinator strips were seeded at a rate of 22 kg/ha using a planter to plant 6 m by 305 m strips (approximately 0.2 ha) directly adjacent to the corn fields. These were planted by late May as soon as field conditions were suitable following corn planting. At each pollinator strip, four honey bee hives were maintained for the duration of the experiment according to local beekeeping practices. Pollinated crops such as flax, canola, and sunflower are uncommon in the study region, and to our knowledge there were no fields located within 3 km of our study locations. Throughout the growing season, honey bees were observed to actively forage in the pollinator strips adjacent to their hives. Thus, the majority of resources brought back to the hives were likely from the pollinator strips as opposed to other mass-flowering crops.

### Sample collection

The most abundant flowering plant species that bloomed across most or all of the study region were selected for clothianidin analysis. In 2014, this included buckwheat, mustard, partridge pea, phacelia, safflower, and sunflower. In 2015, these species were buckwheat, crimson clover, mustard, partridge pea, phacelia, plains coreopsis, safflower, and sunflower. Although mustard was excluded from the seed mix in 2015, wild mustard grew as a weed at organic sites, and was included for analysis in both years.

Leaf tissue (4.5 mm leaf discs) was collected from five different plants at each site when the plant was flowering. Honey and bee bread samples (n = 5 cells each) were collected twice in 2015 from each site when the pollinator strips started to bloom, and again when the pollinator strips were finished flowering in the first week of September (n = 10 samples total per site). Nectar was obtained from foraging honey bees after they were observed landing on a particular flower species. All honey bees on a particular species were tapped into a cup containing 70% ethanol, and the crop removed for clothianidin analysis. Due to the high forage fidelity of honey bee workers^42^, we can assume that all nectar recovered in the crop came from the same species on which the bee landed. All samples were frozen at −20 °C until analysis.

### Clothianidin analysis

Enzyme-linked immunosorbent assays (ELISAs) were used to detect clothianidin in plant samples (product no. 500800, Abraxis, Warminster, PA). Thiamethoxam metabolizes to clothianidin in plants^56^, thus a test for total clothianidin would therefore capture uptake of both seed treatment chemistries. This technique provides an affordable and highly sensitive means of rapidly quantifying concentrations in environmental samples. The test also detects imidacloprid, a chemistry absent from the study sites, with negligible cross-reactivity with other neonicotinoid compounds. ELISAs were conducted according to manufacturer instructions.

Individual leaf discs were homogenized in 258 μl of ddiH_2_0 with a sterile plastic pestle, placed on a shaker for 1 h, and centrifuged at 16,100 g for 3 min. The supernatant was diluted to 20% prior to analysis. In order to control for any plant matrix effects, the species being analyzed were also grown in the greenhouse free from neonicotinoid exposure. Leaf extract from these control plants was spiked with clothianidin (product no. 33589, Sigma-Aldrich, St. Louis, MO) to create a 20% leaf extract with known concentrations for the standard curves^18^,^36^.

Pollen from the bee bread samples (0.05 g) was germinated in 2 ml of a 10% sucrose/0.1% boric acid solution for 2 h at 23 °C^57^. This solution was centrifuged, and the supernatant used in analysis without further dilution. Quality control checks using clothianidin-spiked corn pollen from untreated plants grown in the greenhouse revealed no interference of the pollen matrix with the ELISA. As a result, water was used as the standard curve matrix.

Honey samples (0.2 g) were individually diluted in 1 ml of water. Matrix interference from waxes, pigments, and carbohydrates has been previously documented to occur in ELISAs analyzing honey^58^, so in our assays the honey solutions were diluted 20-fold prior to analysis. Water was used as the matrix in the standard curves.

Nectar was extracted from the crops of honey bees collected foraging in the conservation strips. The crops of multiple bees were combined until the 50 μl of nectar necessary for analysis was obtained. These samples were homogenized using a sterile plastic pestle and centrifuged (16,100 g for 3 min) to remove membranes and any particulates present in the crop. Nectar was used in ELISA analysis without further dilution with water in the standards.

In order to determine whether a sample was positive, that sample’s absorbance was compared to the average absorbance of the controls on the same plate, multiplied by twice the standard deviation of those controls^18^. If the absorbance was less than this value, the sample was considered positive, and compared to the absorbance of standard concentrations of 0, 0.03125, 0.0625, 0.125, 0.25, 0.5, 1, and 2 μg clothianidin/L (ppb). All standard curves were run in triplicate on each plate. For all samples, absorbance was recorded at 450 nm using a μ Quant (Biotek Instruments, Winooski, VT) microplate reader.

### Nutrient analyses

Honey bee adults were collected from the base of the hives at each location every two weeks for 10 weeks and stored in 70% EtOH at −20 °C until analysis. The legs and gut were removed, and the remaining carcass (n = 10 per site per sampling date) homogenized in 100 μl of 1% PBS with a sterile plastic pestle. A 15 μl aliquot for each specimen was then added to 300 μl of methanol-chloroform (2:1 solution) and centrifuged at 16,100 g for 4 min (following Pumariño, *et al*.^32^). The supernatant was transferred to a glass tube for the phosphovanillin lipid assay (modified from Vanhandel^59^), and the pellet retained for the anthrone glycogen assay (modified from Vanhandel^60^). A separate 10 μl aliquot was diluted to 10% with ddiH_2_O for analysis with the Bradford protein assay^61^ (Bio-Rad protein assay kit II, product no. 500-0002) following the manufacturer’s instructions for microplate assays.

Lipid, glycogen, and protein content per honey bee were calculated from the absorbance values (measured at 525 nm [lipids], 630 nm [glycogen], and 595 nm [protein]) using a standard curve. Standard materials were extra virgin olive oil (54 μl olive oil in 50 ml chloroform), glycogen from oyster (25 mg oyster type II [Sigma-Aldrich] in 25 ml water), and protein assay standard II (bovine serum albumin) for lipids, glycogen, and protein, respectively. Standard concentrations were 0, 1, 5, 10, 25, 50, 75, and 100 μg for lipids and glycogen and 0, 25, 50, 100, 250, and 500 μg for protein. All samples and standards were analyzed in triplicate.

### Statistical analysis

Data were analyzed using JMP Pro v.12.1.0 (SAS Institute, Cary, NC). Because data upheld assumptions of normality and homogeneous variances, no transformations were necessary. Leaf data were analyzed using a linear mixed model approach to control for sample independence, with the model testing for differences in clothianidin accumulation between plant species, treatment (organic versus seed-treated locations), and an interaction between these factors, with the unit of replication being plant species per site-year. The random effect was the site-year. Similarly, clothianidin accumulation in bee bread and honey were analyzed using a linear mixed model, with site being the unit of replication and factors being sample date, treatment, and an interaction. Linear mixed model analysis was also used to analyze the nectar data. Because samples from individual bees were pooled in order to obtain an adequate volume for analysis, there was unequal replication between sites, thus the unit of replication became the individual sample and an analysis of organic vs. seed treated treatments was not possible. In order to determine whether there was a difference between the likelihood of encountering contaminated forage at conventional and organic sites, a z-test of clothianidin-positive leaf samples was used. Total glycogen, lipids, and protein were averaged for each site-year and regressed against the clothianidin concentrations in bee bread and honey to quantify any effects of forage exposures to overall health.

## Additional Information

**How to cite this article**: Mogren, C. L. and Lundgren, J. G. Neonicotinoid-contaminated pollinator strips adjacent to cropland reduce honey bee nutritional status. *Sci. Rep*. **6**, 29608; doi: 10.1038/srep29608 (2016).

## Supplementary Material

Supplementary Information

## Figures and Tables

**Table 1:** The proportion of leaf disc samples that tested positive for clothianidin.

	Buckwheat	Mustard	Partridge Pea	Phacelia	Plains Coreopsis	Safflower	Sunflower
Conventional	0.94 (35)*	0.25 (20)	0.60 (20)	0.63 (30)	1.0 (10)	0.86 (29)	0.76 (34)
Organic	0.95 (40)	0.23 (30)	0.60 (15)	0.76 (29)	1.0 (5)	0.82 (39)	0.77 (35)
z-statistic	0.19	0.24	0	1.0	0	0.44	0.10
P	0.85	0.81	1.0	0.32	1.0	0.66	0.92

^*^Total samples analyzed in parentheses.

**Table 2:** Plant species included in the conservation strip mixes for both years.

Common name	Species	% by wt. 2014	% by wt. 2015
Mancan buckwheat	*Fagopyrum esculentum*	48.4	26.8
Finch safflower	*Carthamus tinctorius*	14.5	16.1
Crimson clover	*Trifolium incarnatum*		12.1
Shoshone sainfoin	*Onobrychis viciifolia*	12.1	10.7
Balady Berseem clover	*Trifolium alexandrinum*		8.1
South Dakota common alfalfa	*Medicago sativa*	7.2	4.0
Peredovik sunflower	*Helianthus annuus*	4.8	7.5
Phacelia	*Phacelia tanacetifolia*	3.4	2.7
Medium red clover	*Trifolium pratense*		5.4
Alsike clover	*Trifolium hybridum*	2.9	
Partridge pea	*Chamaecrista fasciculata*	2.9	1.6
Sweet clover	*Melilotus officinalis*		2.7
Ladino clover	*Trifolium repens*	1.2	
Mustard	*Sinapis alba*	1.2	
Indian blanketflower	*Gaillardia aristata*	0.7	0.7
Perennial lupine	*Lupinus perennis*		0.5
Black eyed Susan	*Rudbeckia hirta*	0.2	0.3
Plains coreopsis	*Coreopsis tinctoria*		0.2
Prairie coneflower	*Ratibida columnifera*	0.2	0.2
Prairie cinquefoil	*Potentilla arguta*		0.1
