# Supplementary material for: Neonicotinoid-contaminated pollinator strips adjacent to cropland reduce honey bee nutritional status
Source: Sci Rep. 2016 Jul 14;6:29608. doi: 10.1038/srep29608 (PMC4944152; doi:10.1038/srep29608)
Supplement: Supplementary Information [file srep29608-s1.pdf]

Supplementary Table S1

Title: Neonicotinoid-contaminated pollinator strips adjacent to cropland reduce honey bee nutritional status

Short title: Neonicotinoids in pollinator strips reduce bee health

Christina L. Mogren[1,2*] and Jonathan G. Lundgren[1,3]

[1]USDA-ARS, 2923 Medary Ave, Brookings, SD 57006, USA

[2*]Corresponding author: *Current address*: Louisiana State University AgCenter, 404 Life Sciences, Baton Rouge, LA 70803, USA

Ph: 225-578-1817

Email: cmogren@gmail.com

[3]*Current address*: Ecdysis Foundation: Blue Dash Farm Initiative, 46958 188th St, SD, 57234, USA

Supplementary Table S1. Additional descriptive information for the field sites.

Site ID	Type	Year	Corn Field Size (ha)	Trade Name	Common Name	Other Adjacent Crop[a]	Previous Crop[b]	Distance to Conventional Crops (m)
C1	Conventional	2014	15	Poncho	Clothianidin	Wheat	Wheat	
C2	Conventional		18	Cruiser	Thiamethoxam	Wheat	Wheat	
C3	Conventional		16	Acceleron	Clothianidin	Pasture	Corn	
C4	Conventional		12	Cruiser	Thiamethoxam	Pasture	Alfalfa	
O1	Organic		18			Pasture (O)	Millet	370
O2	Organic		23			Alfalfa (O)	Alfalfa	230
O3	Organic		5.6			Corn (C)	Field peas	10
O4	Organic		12			Hay (O)	Alfalfa	380
C5	Conventional	2015	14	Poncho	Clothianidin	Corn	Soybeans	
C6	Conventional		11	Cruiser	Thiamethoxam	Wheat	Hay	
C7	Conventional		59	Cruiser	Thiamethoxam	Peas/Oats	Corn	
C8	Conventional		27	Acceleron	Clothianidin	Pasture	Soybeans	
O5	Organic		21			Pasture (O)	Soybeans	140
O6	Organic		19			Pasture (C)	Corn	350
O7	Organic		16			Soybeans (O)	Soybeans	200
O8	Organic		19			Pasture (C)	Soybeans	175

[a]Pollinator strips were all planted parallel to corn fields. Adjacent crop refers to the land use on the other side of the pollinator strip. At organic sites, (C) refers to a conventionally managed land and (O) refers to organically managed land.

[b]The crop planted in the corn field in the previous year.
